# HopW1 from *Pseudomonas syringae* Disrupts the Actin Cytoskeleton to Promote Virulence in Arabidopsis

**DOI:** 10.1371/journal.ppat.1004232

**Published:** 2014-06-26

**Authors:** Yongsung Kang, Joanna Jelenska, Nicolas M. Cecchini, Yujie Li, Min Woo Lee, David R. Kovar, Jean T. Greenberg

**Affiliations:** Department of Molecular Genetics and Cell Biology, The University of Chicago, Chicago, Illinois, United States of America; Michigan State University, United States of America

## Abstract

A central mechanism of virulence of extracellular bacterial pathogens is the injection into host cells of effector proteins that modify host cellular functions. HopW1 is an effector injected by the type III secretion system that increases the growth of the plant pathogen *Pseudomonas syringae* on the Columbia accession of Arabidopsis. When delivered by *P. syringae* into plant cells, HopW1 causes a reduction in the filamentous actin (F-actin) network and the inhibition of endocytosis, a known actin-dependent process. When directly produced in plants, HopW1 forms complexes with actin, disrupts the actin cytoskeleton and inhibits endocytosis as well as the trafficking of certain proteins to vacuoles. The C-terminal region of HopW1 can reduce the length of actin filaments and therefore solubilize F-actin *in vitro*. Thus, HopW1 acts by disrupting the actin cytoskeleton and the cell biological processes that depend on actin, which in turn are needed for restricting *P. syringae* growth in Arabidopsis.

## Author Summary

Eukaryotic cells require a dynamic actin cytoskeleton for basic functions, some of which are important for immune responses. Such functions include the transport of cellular material to and from different cellular compartments. The plant pathogen *Pseudomonas syringae* is extracellular and causes disease by injecting effector proteins into plant cells. One of these effectors is HopW1, which disrupts the actin cytoskeleton and reduces the transport of vesicles from the cell surface and proteins destined for vacuoles. The effects of HopW1 can be mimicked using a drug that inhibits actin polymerization. Thus, this work establishes a direct mechanism for pathogen disruption of the actin cytoskeleton and implicates actin-dependent events as important for controlling pathogen growth during infection.

## Introduction

Plants that are infected with foliar bacterial pathogens can mount a multilayered response, the success of which is shaped by the perception of pathogen-derived molecules and the ability of the pathogen to disrupt host responses. Essential for understanding dynamic host-pathogen interactions is the identification of critical components of the host defense machinery and the biochemical mechanism by which bacterial factors interfere with host functions. At least two types of molecules from plant pathogenic bacteria can trigger defenses: conserved patterns (pathogen-associated molecular patterns, PAMPs) that bind to cell surface pattern receptors and more variable effectors that are injected by bacteria into the plants [1]. The perception by plants of some bacterial effectors occurs through the deployment of intracellular immune complexes [1]. A major consequence of bacterial effector activities is to promote virulence, which can occur when plants lack immune receptors for particular effectors. Some of the best-studied effectors are those that form the set of proteins introduced into plants through a type three secretion system (TTSS) [2].

*Pseudomonas syringae* is an extracellular pathogen that causes several types of foliar disease in agriculturally important plant species [3]. In the model plants *Arabidopsis thaliana* and *Nicotiana benthamiana*, pattern receptors and hormonal signals contribute quantitatively to defense against virulent *P. syringae* [1], [4], [5]. However, some *P. syringae* effectors can inhibit the action of receptors, accumulation and/or action of hormone/defense signals and other processes important for quantitative defense activation [6], [7], [8].

An emerging area of research has focused on cytoskeleton components as specific virulence targets of *P. syringae*. At least one effector, HopZ1a, acetylates tubulin *in vitro* and causes microtubule disruption *in planta* [9]. Treatment of plants with cytochalasins, compounds that prevent actin polymerization, increases the ability of several fungal pathogens to penetrate plant tissue [10], [11]. Infection of Arabidopsis with *P. syringae* or treatment with PAMPs induces dynamic changes in actin filament density and bundling [12], [13]. Application of a drug that depolymerizes filamentous actin (F-actin) causes increased growth of *P. syringae in planta* [12]. These observations raise the possibility that specific effectors target the actin cytoskeleton to disrupt actin-dependent immune responses. In plants, the actin cytoskeleton is important for various cell biological processes [14] that may be important for immune signaling, including endocytosis and the trafficking of some vacuolar proteins [15]. While specific *P. syringae* effectors that target actin have not yet been reported, injected virulence factors from several mammalian pathogens have been shown to directly interact with actin or modify cellular components that regulate actin [16], [17], [18].

Nearly a decade of research on effectors has uncovered several examples of *P. syringae* TTSS effectors that can interact with multiple host proteins. Whereas some interactions trigger immunity, others promote virulence [8], [19]. The HopW1 gene, which resides on a multicopy plasmid in *P. syringae* pv. *maculicola* strain ES4326 [20], is an example of an effector that, when expressed in *P. syringae* pv. *tomato*, triggers strong immunity in the Arabidopsis Ws accession and *N. benthamiana*, but promotes virulence in the Arabidopsis Col accession [21]. HopW1 harbors an N-terminal domain that is also found in the N-terminal region of HopAE and a C-terminal domain found in a predicted effector from pathogenic *E. coli* [22], both of unknown functions. The C-terminal domain of HopW1 is critical for triggering defenses and interacting with three HopW1-interacting proteins (WIN1, WIN2 and WIN3) [21].

In the present study, we focus on a virulence function of HopW1. Here, we identified actin as a major component of HopW1-containing complexes purified from plants. We show that F-actin is a major virulence target of HopW1 that *in vitro* disrupts actin filaments and *in planta* disrupts the actin cytoskeleton and interferes with actin-dependent cell biological processes important for plant immunity.

## Results

### Actin Co-purifies with HopW1

**Figure 1:**
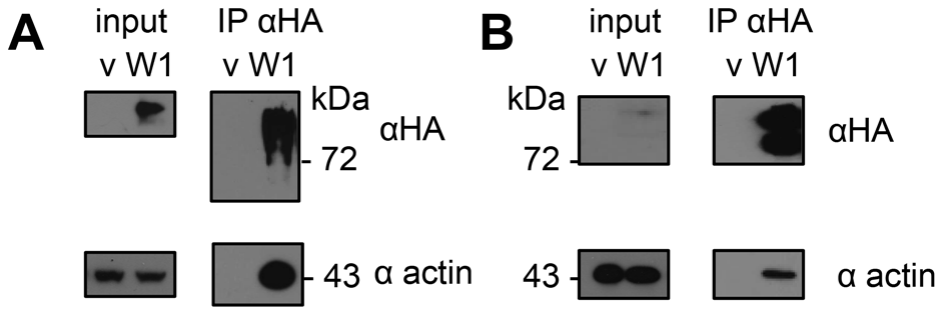
HopW1 forms complexes with actin in plants. Actin was detected by immunoblotting after immunoprecipitation (IP) of HopW1-HA complexes. (A) HopW1-HA-actin complexes in *N. benthamiana* transiently transformed with HopW1-HA (W1) using Agrobacteria. V is vector control. (B) HopW1-actin complexes in dexamethasone (dex)-treated Arabidopsis stable transgenics that carry dex:HopW1-HA. Input was 2% of extract used for each IP. These experiments were each repeated twice with similar results.

To find components of HopW1-containing complexes, we used LC-MS/MS to identify proteins that co-purified with HopW1-HA that was transiently expressed in *N. benthamiana*. Thirteen unique peptides derived from actin (covering 47% of protein) were identified from a co-precipitating band of 43 kDa that was absent in the control immunoprecipitation (IP). We confirmed that HopW1 and actin formed a complex using IP and immunoblotting of extracts from *N. benthamiana* and Arabidopsis that transiently expressed HopW1-HA (Figure 1). HopW1 does not have any know actin binding motifs or sequences that could help predict its activity. However, the high amount of actin in HopW1 complexes prompted us to investigate HopW1 influence on the actin cytoskeleton.

### HopW1 Disrupts Actin Filaments during Infection

**Figure 2:**
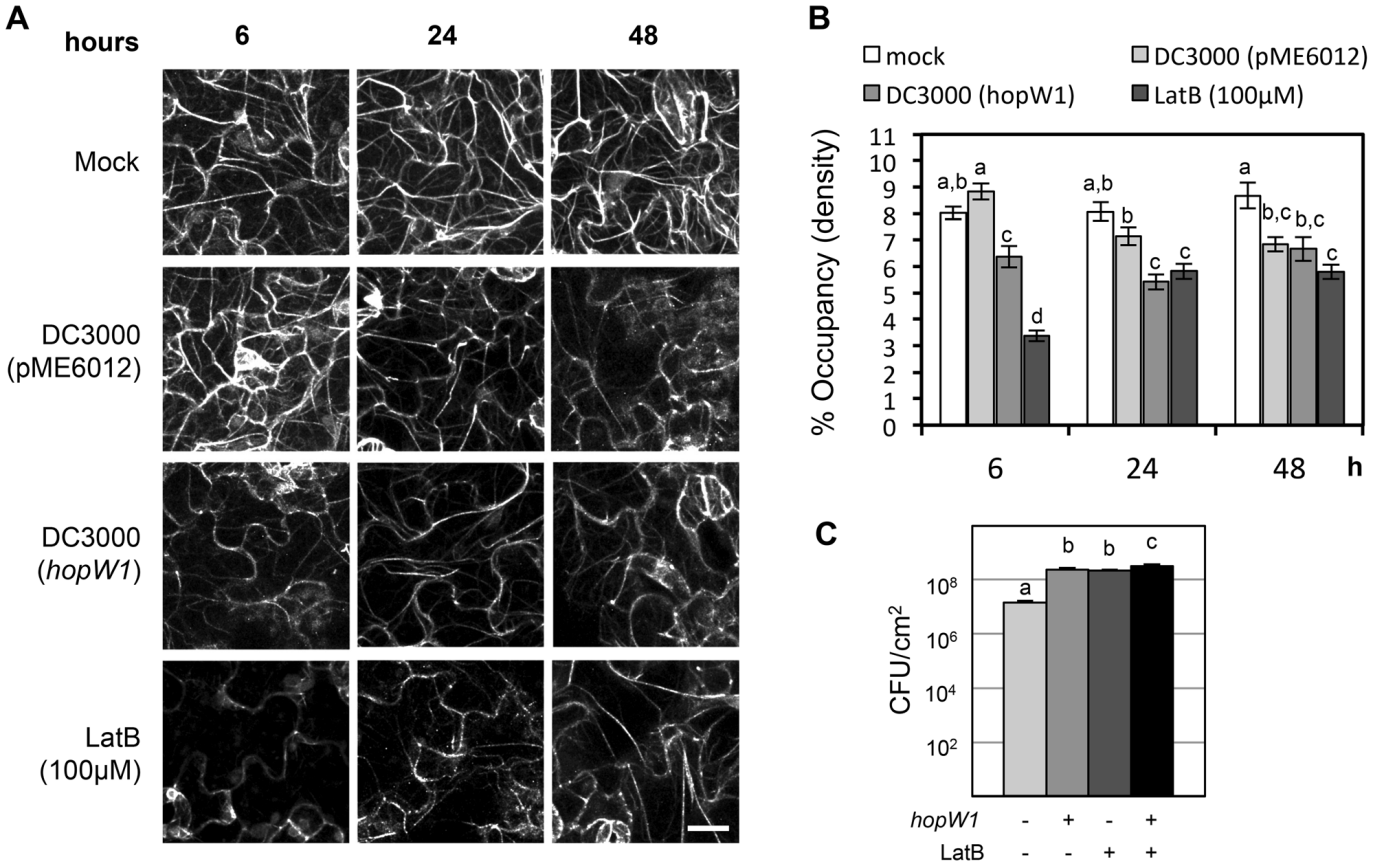
HopW1 disrupts the actin cytoskeleton during infection and promotes *Pto*DC3000 growth on Arabidopsis. (A) Actin cytoskeleton changes in cotyledons after infection with *Pto*DC3000/HopW1. Col/Lifeact-GFP [26] seedlings grown on MS plates were infected with *Pto*DC3000/empty vector or *Pto*DC3000/HopW1 at OD_600_ = 0.01 or treated with 100 µM LatB (actin cytoskeleton-disrupting control). Cotyledons were imaged by laser-scanning confocal microscopy at 6, 24 and 48 h after infection/treatment. Representative micrographs shown are Z-series maximum intensity projections. Bar = 18 µm (B) Percent occupancy (density) of actin filament signals shown in (A) was measured using ImageJ software as described in [12], [25] in 18 samples per treatment/time-point, from three biological repeats from picture regions without stomata. (C) Growth of *Pto*DC3000/vector or *Pto*DC3000/HopW1 (OD_600_ = 0.0001) three days post-inoculation of 3-week old soil grown Arabidopsis Col leaves was monitored in the presence or absence of LatB. These experiments were performed three times with similar results. In B and C, average of all results is shown, different letters indicate significant difference (ANOVA/Tukey's test, *P*<0.05, n = 18 in B and n = 9 in C) and bars represent SEM.

In eukaryotic cells, actin exists as both dynamic filaments (F-actin) and as a large pool of unpolymerized actin [23]. We used Arabidopsis Col expressing Lifeact-GFP (green fluorescent protein) that binds F-actin [24], [25], [26] to visualize the actin cytoskeleton (Figure 2A). We imaged F-actin by confocal microscopy in Col/Lifeact-GFP seedlings infected with *Pto*DC3000 containing HopW1 (*Pto*DC3000/HopW1) or a vector control (pME6012). We used ectopic expression in a heterologous *Pto* strain that does not a contain a HopW1 homologue, since we were not able to delete HopW1 from its native strain *Pma*ES4326, where HopW1 resides on a large multicopy plasmid [20], [21]. Ectopic HopW1 expressed in *Pto*DC3000 is functional: it confers increased pathogen virulence in Arabidopsis Col and reduced virulence in Ws, where HopW1 strongly triggers defenses [21]. Actin filament density was quantified as the percentage of Lifeact-GFP signal occupancy (or density) in confocal micrographs, as described in [12], [25]. We observed a trend of a transient increase of F-actin density in cotyledons at 6 h after *Pto*DC3000/vector infection and a later decrease at 48 h compared with a mock treatment (Figure 2B), similar to a previous report [12]. *Pto*DC3000/HopW1 infection or treatment with an inhibitor of actin polymerization, latrunculin B (LatB) caused a decrease in F-actin already after 6 h that persisted also at later time (24 and 48 hpi). The actin cytoskeleton was significantly more disrupted during infection with *Pto*DC3000/HopW1 than *Pto*DC3000/vector at 6 and 24 h (Figure 2B). Thus, HopW1 causes an early disruption of the actin cytoskeleton during infection.

### The Virulence Effect of HopW1 Is Phenocopied by Latrunculin B

If the actin cytoskeleton is a major target of HopW1, a prediction is that pharmacological disruption of the actin cytoskeleton should phenocopy the virulence effect of HopW1. Indeed, LatB treatment increased the growth of *Pto*DC000 on Arabidopsis [12], an effect we also observed (Figure 2C). LatB had the same magnitude of effect as HopW1 to increase *Pto*DC3000 growth on Col Arabidopsis (Figure 2C). *Pto*DC3000/HopW1 grew slightly more than *Pto*DC3000/vector in LatB-treated plants. However, the additive effect was very small and might not be biologically meaningful. The results show that the net effects of HopW1 and LatB are similar, consistent with disruption of actin being responsible for increased pathogen growth.

### HopW1 Alone Disrupts the Actin Cytoskeleton in Plant Cells

**Figure 3:**
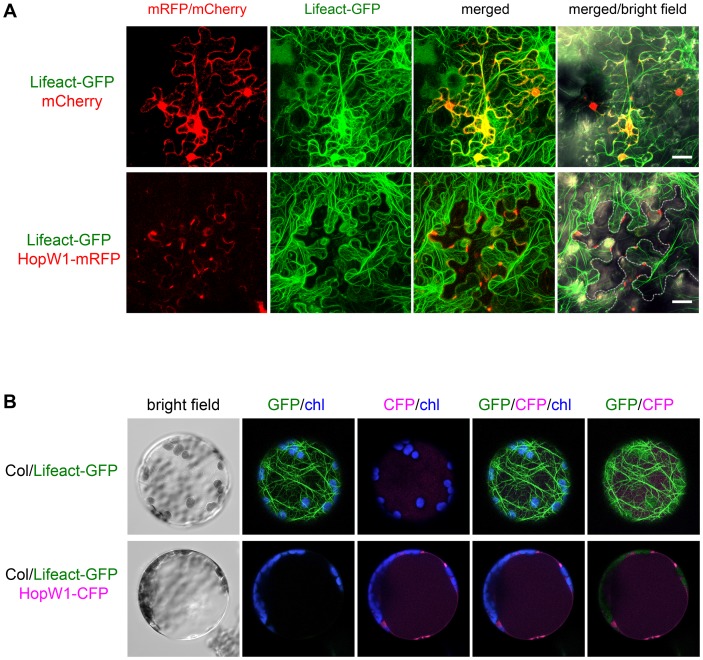
HopW1 disrupts the actin cytoskeleton when expressed in plant cells. Localization and effect of HopW1 on actin cytoskeleton was monitored in transiently transformed cells using laser scanning confocal microscopy. Representative micrographs shown are Z-series maximum intensity projections. (A) Expression of HopW1-RFP and Lifeact-GFP F-actin marker in *Nicotiana benthamiana* leaves 36–40 h after co-transformation with Agrobacteria. Micrographs show localization of cytoplasmic mCherry (control, upper panel) and HopW1-RFP (lower panel) together with Lifeact-GFP. GFP/RFP fluorescence is shown in green/red, respectively. Dotted line shows cells expressing HopW1-RFP. Bar = 30 µm. (B) Arabidopsis Col protoplasts from a transgenic line that expresses Lifeact-GFP [26] were transfected with HopW1-CFP (lower panel) or without DNA (control, upper panel). Micrographs show Lifeact-GFP and HopW1-CFP 15 h after transfection. GFP/CFP fluorescence is shown in green/magenta, respectively, and chloroplast (chl) autofluorescence in blue. These experiments were repeated twice with similar results. The actin cytoskeleton was not detectable in all cells in which HopW1-RFP/CFP was observed (at least 30 *N. benthamiana* cells and 40 Lifeact-GFP Arabidopsis protoplasts, respectively, with HopW1 signal were observed).

We directly expressed HopW1 in plant cells to see if it is sufficient to affect the actin cytoskeleton. When transiently co-expressed in *N. benthamiana* with Lifeact-GFP, HopW1-RFP (red fluorescent protein) disrupted F-actin to such a degree that HopW1 was only detected in cells with very little Lifeact-GFP 36–40 h after Agroinfiltration (Figure 3A). This pattern was seen in all cells with detectable HopW1. At earlier times (16–24 h after Agroinfiltration), no fluorescent protein signals were detected. Plant cells in which the actin cytoskeleton was labeled with Lifeact-GFP lacked detectable HopW1-RFP signals. In contrast, dense F-actin was present in cells with control mCherry (Figure 3A). When detectable, HopW1-RFP accumulated in patches at the periphery of cells (Figure 3A). Similar patterns were observed when HopW1-RFP was transformed alone into *N. benthamiana* leaves without Lifeact-GFP (not shown).

Localization of HopW1 in patches may result from disorganization of the actin cytoskeleton at the time when we can detect HopW1. During the infection of Arabidopsis with *Pto*DC3000/HopW1 or LatB treatment, most Lifeact-GFP marked filaments also disappeared from the cell interiors and the signal remained at the periphery (Figure 3A). Similarly, in protoplasts from Arabidopsis Lifeact-GFP plants transiently expressing HopW1-CFP (cyan fluorescent protein), the F-actin cytoskeleton was absent 15 h after transformation and HopW1-CFP was found in patches mostly along cell border (Figure 3B). Only protoplasts with undetectable HopW1-CFP signal had an intact actin cytoskeleton (not shown). These data show that HopW1 is sufficient to disrupt actin filaments in plant cells.

### HopW1-C Disrupts F-actin *In Vitro*

**Figure 4:**
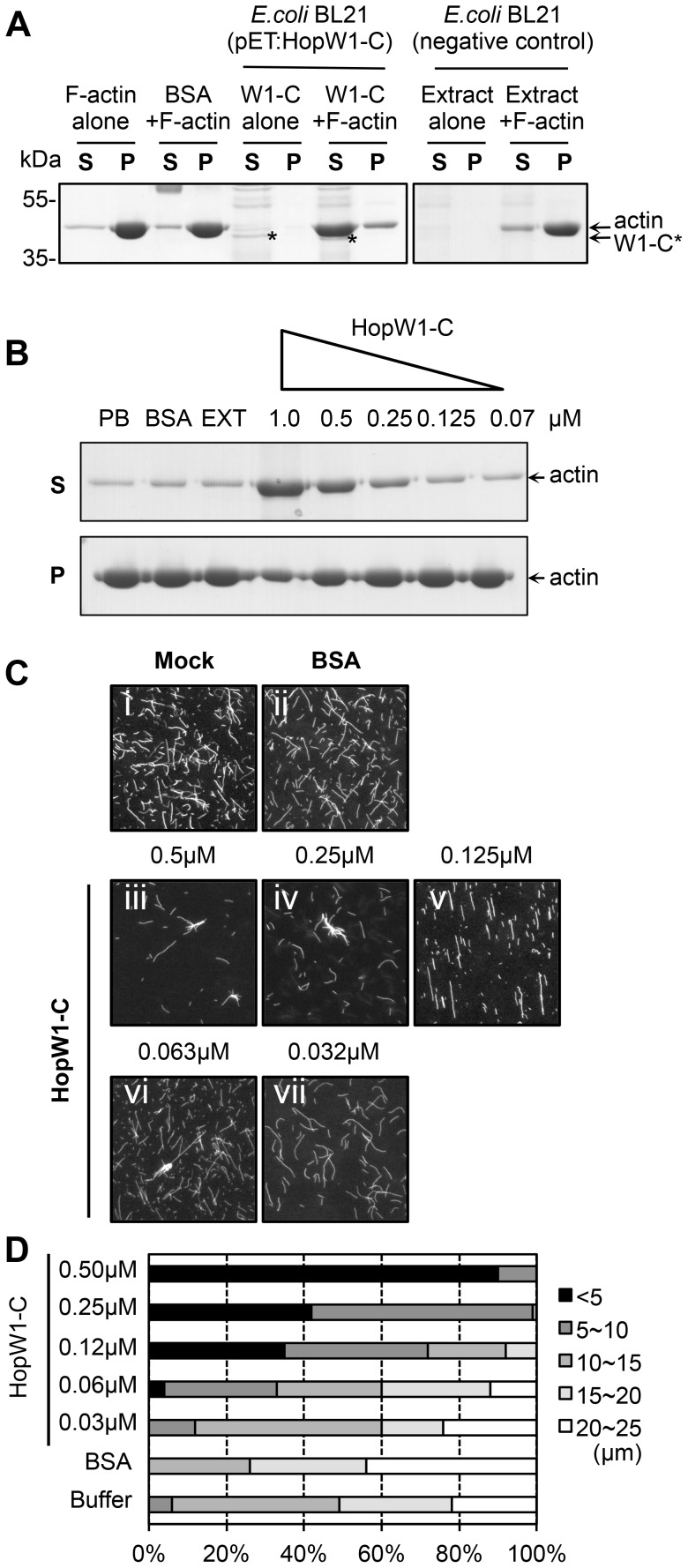
HopW1-C disrupts F-actin *in vitro*. (A) HopW1-C (HopW1^407–774^) causes a reduction in the size of actin filaments *in vitro* as assayed by sedimentation ultracentrifugation. Non-muscle F-actin (10 µM) was incubated with HopW1-C (W1-C, asterisks) for 30 min, partitioned into supernatant (S) and pellet (P) fractions by ultracentrifugation, separated by SDS-PAGE and stained with Coomassie blue. Greater than 90% of actin was found in the pellet in the absence of HopW1-C. In contrast, after incubation with 0.5 µM HopW1-C, >70% of actin was in the supernatant (note concomitant decrease of actin in the pellet). (B) F-actin disruption by HopW1-C is dose dependent. Preassembled F-actin (10 µM) was disrupted in the presence of different amounts of HopW1-C, but not with the controls: phosphate buffer (PB), BSA, and *E. coli* BL21 extract (EXT) after 30 min incubation. (C) Visualization of F-actin disruption. Mock (50 mM phosphate buffer) (i), BSA (0.5 µM) (ii), and different amounts of HopW1-C (iii to vii) were incubated with 5 µM F-actin for 1 h. Filaments were stained with TRITC-phalloidin and observed by epifluorescence microscopy. (D) Quantitation of the reduced F-actin lengths. Actin filaments (≥100) from (C) were measured in each treatment. The distribution of lengths of F-actin was different for 0.03 µM to 0.5 µM HopW1-C (W1-C) treatments compared with buffer control (mock), as determined by *χ* ^2^ tests (*P*<0.0001). These experiments were repeated three or more times with similar results.

To test whether the effect of HopW1 on the actin cytoskeleton observed *in planta* is direct, we assayed the activity of recombinant HopW1 on actin filaments *in vitro*. To evaluate the ratio of soluble to F-actin, we performed sedimentation assays after 30 min. incubation of pre-assembled F-actin with recombinant His-HopW1-C (HopW1^407–774^) [27]. We used a truncated version of HopW1 because full-length protein was insoluble. We validated that actin co-purified from plants with full length and the C-terminal domain, but not with N-terminal domain, although HopW1-C accumulated *in planta* at much lower level than other variants (Figure S1). HopW1-C, but not *E. coli* extract or BSA, increased the amount of actin in the supernatant and simultaneously decreased actin in the pellet (Figure 4A), indicating a dose-dependent ability of HopW1-C to solubilize F-actin (Figure 4B).

Sedimentation assays were corroborated by visualizing changes in the distribution of actin filament lengths after 1 h incubation with a range of HopW1-C concentrations. There was a clear and statistically significant shift to smaller filament lengths as a function of the amount of HopW1-C added to preassembled F-actin (Figure 4C and 4D). These assays employed non-muscle actin. We did not detect disruption of muscle F-actin by HopW1-C (Figure S2). Thus, HopW1-C can directly disrupt non-muscle F-actin *in vitro*.

### HopW1 Disrupts Actin-Dependent Protein Targeting

**Figure 5:**
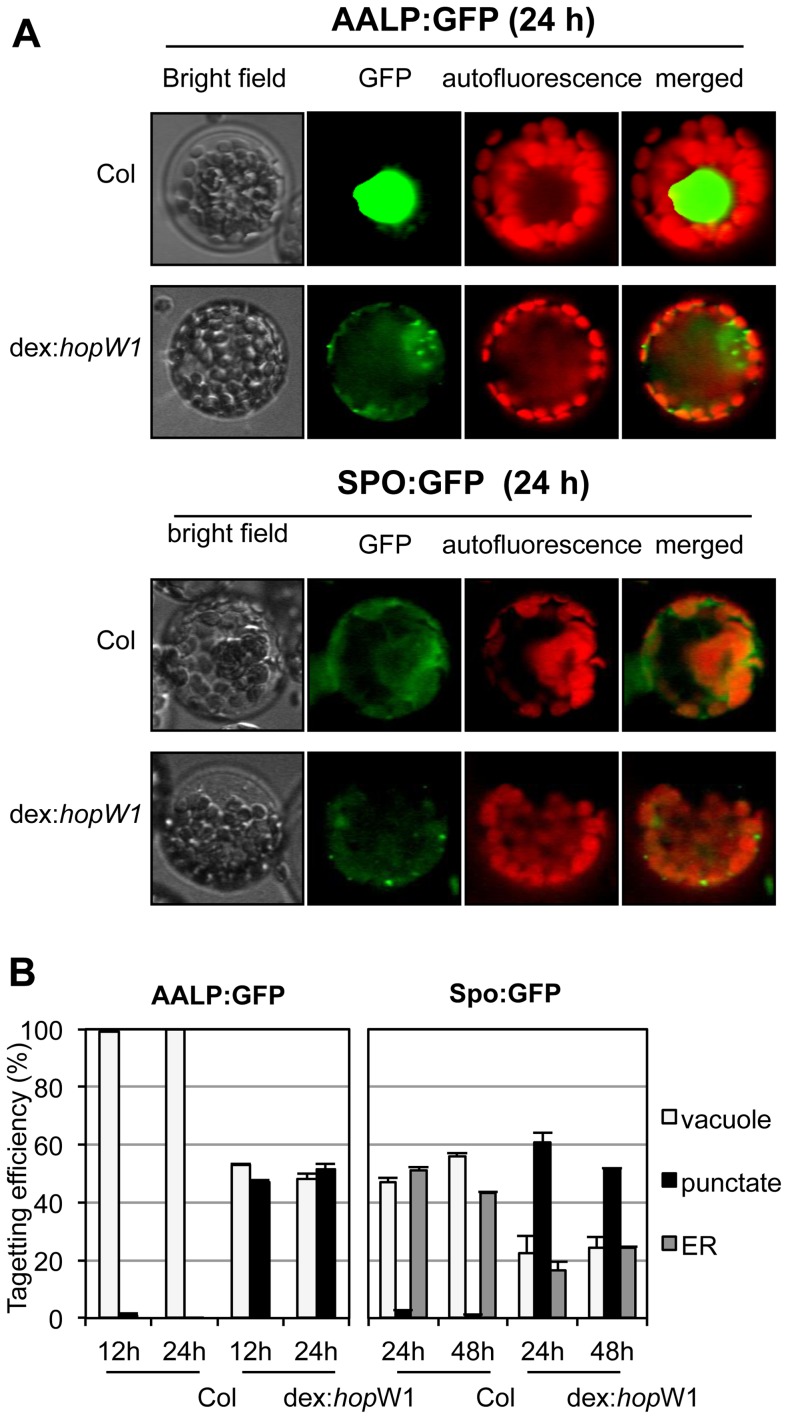
Inhibition of the vacuolar and ER trafficking by HopW1. (A) Wild-type and dex:*hopW1* Arabidopsis Col protoplasts were transfected with AALP:GFP or SPO:GFP and incubated with 0.2 µM dex for the indicated times and imaged using fluorescence microscopy. AALP:GFP was targeted to the central vacuole and SPO:GFP localized to the ER and vacuole in wild-type protoplasts, as previously documented [14]; upper rows in each panel show examples of vacuole and ER localization, respectively. However, in the presence of HopW1, many protoplasts transfected with the AALP:GFP and SPO:GFP showed notable punctate patterns. (B) Localization patterns were quantified from at least 100 images, such as those in (A); see also Figure S2A for comparison with the effect of LatB. Bars indicate SEM. *χ* ^2^ tests indicated that the distributions were significantly different between the wild-type and dex:*hopW1* at each time point (*P*<0.0001, n≥100 per genotype/fusion construct). This experiment was performed three times using at least two transgenic dex:HopW1 lines, with similar results.

Disruption of the actin cytoskeleton may lead to disruption of intracellular trafficking that is essential for plant immunity [13], [28], [29], [30]. As a first test of this possibility, we assessed HopW1's effect on the trafficking of marker proteins. A functional actin cytoskeleton is needed for the trafficking of sporamin-GFP (SPO-GFP) and Arabidopsis aleurin-like protein-GFP (AALP-GFP) to the endoplasmic reticulum (ER) and/or vacuole [14] (ER localization of SPO-GFP occurs prior to its transport to the vacuole [14]). In the absence of HopW1, both reporter proteins individually transfected to Col protoplasts showed patterns consistent with ER and/or vacuole localization (Figure 5A, upper panels), as previously shown [14]. In contrast, in transgenic Arabidopsis protoplasts expressing dex-induced HopW1, the normal patterns of SPO-GFP and AALP-GFP signals were disrupted at 12, 24 and 48 h post transformation and HopW1-dependent punctate patterns appeared (Figure 5A, bottom panels). Control treatment with LatB to disrupt the actin cytoskeleton also caused similar punctate GFP patterns (Figure S3A), as previously reported [14]. Quantitation of the GFP patterns showed that HopW1 (and LatB) significantly disrupted the normal localization patterns of AALP-GFP and SPO-GFP, respectively (Figure 5B and S3B). Therefore, HopW1 prevents normal localization of proteins whose targeting depends on actin in a similar way as a drug that disrupts the actin cytoskeleton.

### HopW1 Disrupts Endocytosis

**Figure 6:**
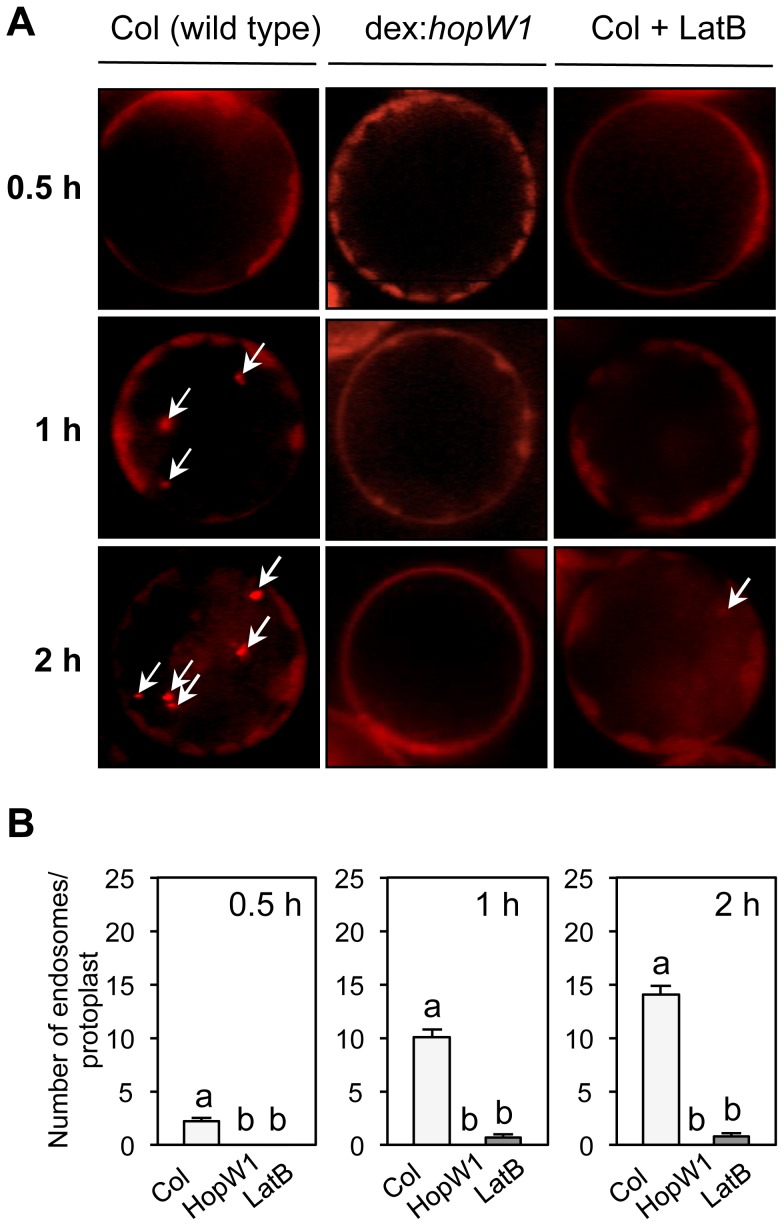
Endocytosis inhibition by HopW1 in Arabidopsis. (A) Representative microscopic images that show the effects of HopW1 and LatB on endocytic vesicle formation. Wild-type and dex:*hopW1* Arabidopsis Col protoplasts were treated with 0.2 µM dexamethasone, stained with FM4-64 and visualized by fluorescence microscopy. 10 µM LatB was used to disrupt the actin cytoskeleton. After over-night incubation with dexamethasone and/or LatB, protoplasts were labeled with FM4-64 and viewed after 0.5, 1 and 2 h. Arrows point to some of the FM4-64-stained endosomes. Protoplasts from two independent transgenic dex:HopW1 lines were used with similar results. (B) Endosomes were quantified in protoplasts from the indicated plants that were treated and stained as in (A). At least 20 protoplasts per treatment, per time-point, from three biological repeats (independent experiments) were analyzed. Bars indicate SEM. Different letters indicate significantly different numbers of endosomes (*P*<0.0001) between wild type versus dex:*hopW1* or LatB-treated wild type, determined using ANOVA/Tukey's test.

A functional actin cytoskeleton is also critical for endocytosis and trafficking of vesicles [23]. We used the lipophilic dye FM4-64 to monitor endocytic trafficking [31] in Arabidopsis protoplasts. Over time (0.5 to 2 h after dye application), increased numbers of endocytic vesicles were stained with FM4-64 in wild-type protoplasts. In contrast, FM4-64 failed to label vesicles in protoplasts from transgenic Arabidopsis expressing dex-inducible HopW1 (Figure 6A and 6B). Endosomes labeled by FM4-64 were also significantly reduced in the LatB-treated versus control protoplasts (Figure 6B). Thus, HopW1 affects actin-dependent cell biological events when directly expressed in plant cells.

### Infection Causes HopW1-Dependent Disruption of Endocytosis

**Figure 7:**
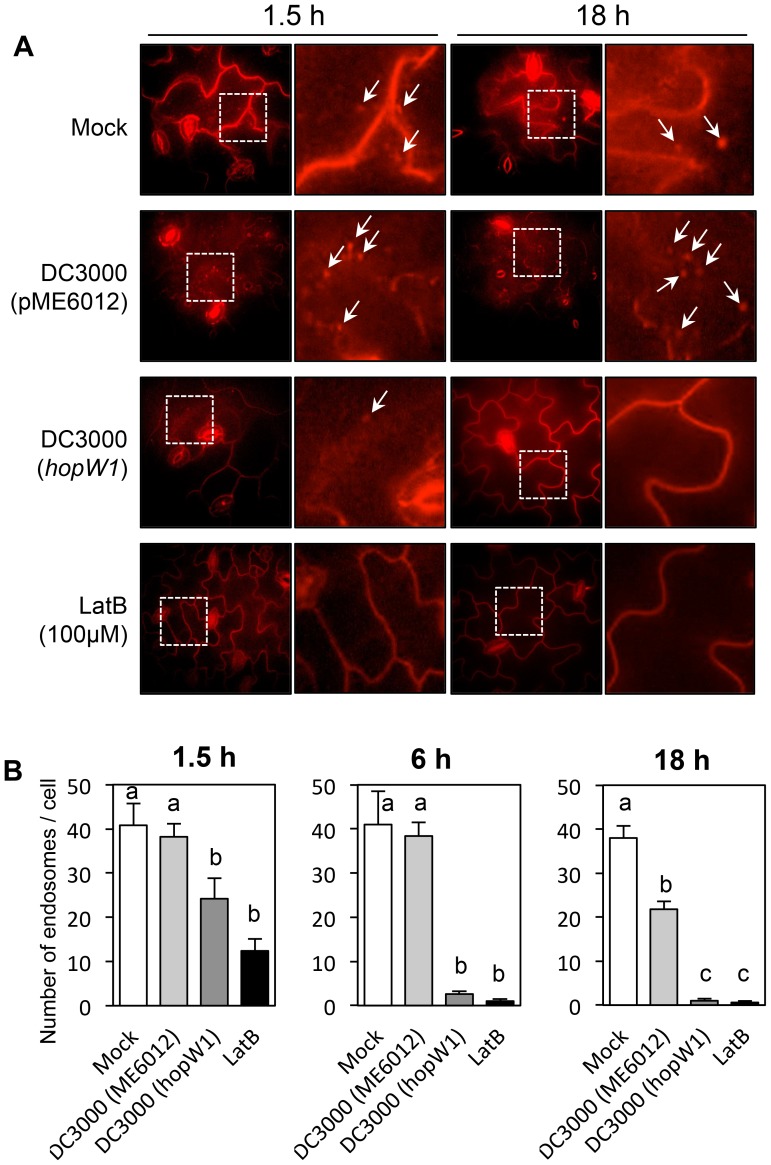
*Pto*DC3000/HopW1 infection inhibits endocytosis. (A) Examples of microscopic images of infected tissue in which endosomes are visualized using FM4-64. Cotyledons of Arabidopsis Col seedlings grown on MS plates were infected with *Pto*DC3000 carrying either empty vector (pME6012) or vector with the HopW1 gene at OD_600_ = 0.01. 100 µM LatB was used as an actin cytoskeleton-disrupting control. After infections and treatments for the indicated times, cotyledons were labeled for 1 h with FM4-64 and viewed. Arrows indicate some of the FM4-64-labeled endosomes. (B) Quantitation of the data in (A). Endosomes per cell were manually counted in at least 10 images per treatment, per time-point, from two or three biological repeats. Bars indicate SEM. Different letters indicate significantly different numbers of endosomes for given treatments, as determined by ANOVA/Tukey's test (*P*<0.05).

We tested whether the delivery of HopW1 during *P. syringae* infection has a similar effect on endocytosis as HopW1 expressed directly in plant cells. To estimate when the inhibition of endocytosis might occur, we infected seedlings with *Pto*DC3000/HopW1 or *Pto*DC3000/vector, or treated seedlings with LatB as a positive control. We monitored endosomes stained with FM4-64 in Col cotyledons 1.5, 6, and 18 h after infection (Figure 7A and 7B). The numbers of endosomes per cell labeled with FM4-64 were highly reduced at 6 and 18 h after infection with *Pto*DC3000/HopW1 and similar to the effect of LatB treatment. In contrast, the number of endosomes was not different from mock treatment in early (1.5–6 h) *Pto*DC3000/vector infection, but was reduced by 50% at 18 h. These results indicate that HopW1 is mainly responsible for the inhibition of endocytosis during early stages of infection, but *Pto*DC3000 may have another factor(s) that also weakly affects endocytosis at the later times. The timing of inhibition of endocytosis by HopW1 is consistent with disruption of actin cytoskeleton during infection (Figure 2).

## Discussion

**Figure 8:**
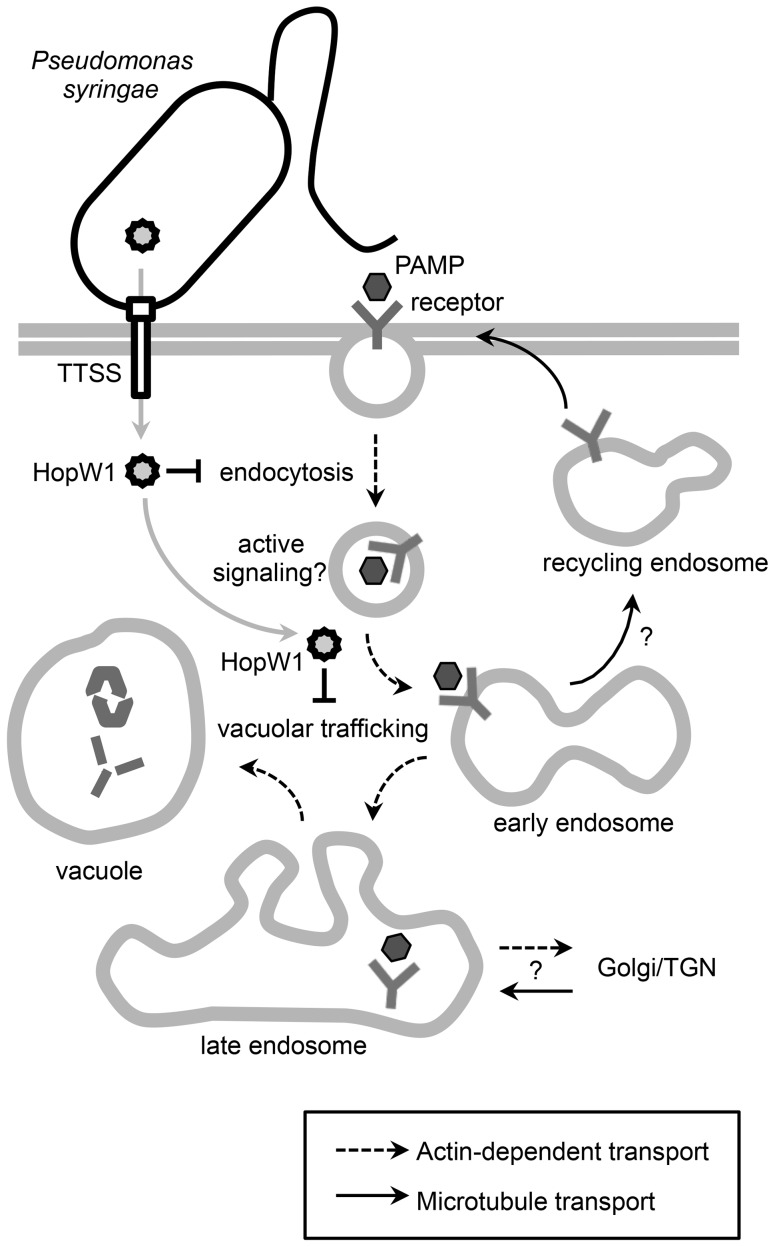
A schematic diagram of HopW1's effects on plant cells. Model showing that the *P. syringae* effector HopW1, secreted through the type three secretion system (TTSS) disrupts actin-dependent events that may be important for defense. Disruption of actin-dependent endocytosis may reduce active signaling from endosomes. It is also possible that disruption of actin-dependent vacuolar trafficking suppresses defenses by preventing efficient delivery of defense proteins to the vacuole or that some other actin-dependent defense process is disrupted. Proteins and possibly other molecules that are targeted to vacuoles have previously been implicated in various defense-related responses to infection [38], [52].

HopW1's virulence activity is strongly linked to its effect on actin and actin-dependent processes in susceptible Col Arabidopsis. Specifically, HopW1 co-purifies with actin from plants and can disrupt F-actin *in vitro* and decrease actin filament density during infection. Figure 8 shows a model for HopW1's possible mode of action as a virulence factor. HopW1 inhibits actin-dependent cell biological processes *in planta*, such as endocytosis and the trafficking of certain proteins destined for ER and/or vacuoles. Surface receptors such as FLS2 or LeEix2 that recognize PAMPs and contribute to basal defense [32] are endocytosed upon activation [33], [34], [35]. Endocytosis of receptors requires an intact actin cytoskeleton [33], but whether endocytosis per se is critical for FLS2 signaling to limit bacterial growth is not clear. Endocytosis of the tomato receptor LeEix2 is important for its immune signaling output [35]. Trafficking to the ER, disrupted by HopW1, is necessary for replacement of endocytosed receptors at the plasma membrane [33], [36] and secretion of antimicrobial factors [37]. Vacuole also has an established role in immunity [38].

HopW1 joins a growing list of pathogenic effectors from infectious bacteria that directly bind actin and/or regulate actin and thus actin-dependent processes [15], [39], [40], [41], [42], [43]. However, HopW1 is the first effector that we know of from a plant bacterial pathogen that directly targets actin. HopW1 can increase the growth of a *P. syringae* isolate that is already relatively successful in growing on Arabidopsis. This implicates an actin-dependent process(es) as important for imparting an immune response to limit the growth of pathogenic bacteria. Disruption of F-actin polymerization with LatB also increases susceptibility of the Col accession of Arabidopsis to *Pto*DC3000 ([12], this paper) confirming the role of actin cytoskeleton in defense. HopW1 is not part of the *Pto*DC3000 effector repertoire, and thus we expect that there may be yet additional effectors from *Pto*DC3000 and/or other *P. syringae* strains that affect actin or actin-dependent processes.

Recently, a study of actin dynamics during infection of Arabidopsis by *Pto*DC3000 implicated effectors as causing increased F-actin bundling late in the infection as part of the virulence mechanism, although no specific effector was identified [12]. We did not notice any effect of HopW1 on F-actin bundling. An early response to PAMPs and infection is an increase in density of actin filaments [12], [13], possibly to assist with higher demand for intracellular trafficking during defense. HopW1 counteracts this response by disrupting F-actin early during infection.

HopW1-C activity *in vitro* is specific for non-muscle (cytosolic) actin. Plant vegetative actin isoforms (expressed in all vegetative organs) are not more phylogenetically similar to non-muscle than muscle animal actins. However, they are functionally more related to cytosolic actins than to specialized muscle actins [44]. Cytosolic actin is essential for processes such as growth and intracellular trafficking in all eukaryotic cells. Our results confirm this functional similarity, recently reported by complementation of Arabidopsis vegetative actin mutants by human and protist cytosolic actins, but not by human muscle actins [44].

Interestingly, the actin-disrupting/actin binding portion of HopW1 (the C-terminal region) is also present in an effector found in some pathogenic *E. coli* strains [22]. A protein BLAST search indicates that the plant pathogen Acidovorax encodes a protein with similarity to the C-terminus of HopW1 (eg. GeneBank accession no. YP 969911). These homologs may also disrupt actin as part of their virulence mechanisms. HopW1 does not have recognizable features corresponding to any known actin depolymerizing or severing factors. However, its localization pattern in patches resembled that of actin-binding proteins involved in F-actin organization at the membrane: class I formins, such as AtFH4 [45] and NET proteins [46]. Future structural analysis may shed light whether HopW1 is an example of a structural mimic of a known activity or whether it disrupts F-actin by a wholly novel mechanism.

## Materials and Methods

### Bacterial Strains and Plasmids

Bacterial strain and plasmids used in this study are listed in Tables S1, S2.

### Bacterial Growth Assays *In Planta*

Bacteria and/or 10 µM LatB were inoculated into leaves of 3-week old *Arabidopsis thaliana* Col accession grown in soil for bacterial growth analyses, which were done as described in Text S1.

### Immunoprecipitation, Western Blot and LC-MS/MS Analysis

HopW1-HA was immunoprecipitated with anti-HA matrix (Roche) from transgenic Arabidopsis or transiently transformed *N. benthamiana* as described Text S1. LC-MS/MS protein identification after trypsin digestion of protein bands was performed at Chicago Biomedical Consortium as described [47]; data was searched against the NCBI database using Mascot and validated with Scaffold 2 (Proteome Software Inc.)

### Evaluation of F-actin Arrays in Cotyledons after Infection

To quantitatively evaluate actin filament populations, we calculated F-actin density in the cell as the percent occupancy of Lifeact-GFP signals based on the captured confocal microscope images. Transgenic Col/Lifeact-GFP [26] cotyledons were infected with *Pto*DC3000/empty vector or *Pto*DC3000/HopW1 at OD_600_ = 0.01 and 100 µM LatB was used as an actin cytoskeleton-disrupting control. Laser-scanning confocal microscopy was used to visualize Lifeact-GFP (see Text S1 for details) in infected epidermal cells. GFP fluorescence from Z-series maximum-intensity projections of 32 optical sections (0.5 µm each) was separated from background by minimal threshold to include all F-actin signals. Images were analyzed by Image J software (http://rsb.info.nih.gov/ij) as described [25]. Gaussian blur and high-band pass filter (1–5 pixels) were applied as described in [12], [25]. Actin filament density was designated as a percentage of total pixel numbers of F-actin (as defined by Lifeact-GFP) per total number of pixels in the photograph [12], [25]. Picture regions without stomata were used for F-actin density calculation.

### Protoplast Isolation and Transient Transformation

Arabidopsis protoplasts isolation, polyethylene glycol (PEG)-mediated transformation [48], [49] and microscopy were done as described in Text S1. Protoplasts from at least two independent transgenic dex:HopW1 lines were used with similar results.

Agrobacterium tumefaciens GV3101 strain harboring pMDC43-Lifeact (F-actin marker) and 35S-mCherry (control) or pGWB454-HopW1 (HopW1-RFP) were co-infiltrated into *N. benthamiana* leaves. 16–40 h after Agro-infiltration, leaves were analyzed by confocal microscopy. At least 30 cells with HopW1-CFP signal were observed in 2 experiments 24–40 h after transformation.

### Monitoring Protein Trafficking in the Presence of HopW1

Constructs for *in vivo* organelle targeting reporter proteins (AALP:GFP and SPO:GFP, kindly provided by Dr. Inhwan Hwang in Pohang University of Science and Technology, South Korea) were transfected into protoplasts derived from Arabidopsis dex:*hopW1* and control non-transgenic plants, and incubated in W5 buffer with 0.2 µM dexamethasone to express HopW1. Protoplasts were monitored using confocal fluorescence microscopy (described in Text S1) at the various times (12 h, 24 h, and 48 h) after transfection/dex treatment. Based on the digital images of transformed protoplasts, we categorized GFP patterns by the distributions of reporters as a vacuolar, an ER, and a punctate pattern, and then we counted and scored the distribution patterns of >100 protoplasts.

### Endocytosis Inhibition Assay in Protoplasts and Cotyledons

Protoplasts prepared from Arabidopsis expressing dex:*hopW1* or non-transgenic controls were incubated overnight with 0.2 µM dexamethasone and then stained with 6.4 µM FM4-64 (Invitrogen, Eugene, OR) for 5 min and washed with W5 buffer. The protoplasts were then incubated for various times (up to 2 h) in W5 buffer at room temperature prior to fluorescence microscopy as described in Text S1.

To study endocytosis after infection, 6- to 8-d old Arabidopsis Col grown on MS agar plates were inoculated by placing a drop of *Pto*DC3000/HopW1 or *Pto*DC3000/vector cultures at OD_600_ of 0.01 in 10 mM MgSO_4_ or 100 µM LatB on each seedling, and incubated in a sterile hood for 30 min to allow the excess liquid to be absorbed by the seedling or evaporate. Seedlings were returned to the growth chamber for various times (up to 24 h). Harvested cotyledons infiltrated with 8.2 µM FM4-64 (Invitrogen, Eugene, OR) using vacuum for 2 min, were incubated 1 h prior to microscopic analyses. Endosomes were manually counted in at least 10 plant cells per treatment per time point.

### Non-muscle F-actin Disruption Assays

F-actin disruption assays followed manufacturer's protocol (Cytoskeleton, #BK013, USA) for sedimentation assays or fluorescence microscopy as described [50], [51] in the presence of purified recombinant HopW1-C (Text S1). Non-muscle actin was polymerized to filaments in 10 mM Tris pH 7.0, 1 mM ATP, 50 mM KCl, 1 mM EGTA, 0.2 mM CaCl_2_ and 2 mM MgCl_2_ for 1 h at room temperature. 10 µM preassembled F-actin was incubated with HopW1-C or controls (BSA or *E. coli* extract) for 30 min. at room temperature and centrifuged at 150,000×g for 1.5 h. Pellet (P) and supernatant (S) fractions were separated by SDS-PAGE.

To examine F-actin disruption by fluorescence microscopy, 5 µM preassembled non-muscle F-actin was incubated with different amounts of HopW1-C or BSA for 1 h at room temperature and stained for 5 min with 1 µM TRITC-Phalloidin (Fluka Biochemika, Switzerland). Reactions were terminated by a 250-fold dilution in fluorescence buffer (50 mM KCl, 1 mM MgCl_2_, 100 mM DTT, 20 µg/ml catalase, 100 µg/ml glucose oxidase, 3 mg/ml glucose, 0.5% methylcellulose, and 10 mM imidazole, pH 7.0) and absorbed to coverslips coated with 0.05 µg/µl poly-L-lysine.

Fluorescence images were collected with a cooled CCD camera (Orca-ER, Hamamatsu) on an Olympus IX-81 microscope. The lengths of at least 100 filaments per treatment were quantified using ImageJ software (http://rsb.info.nih.gov/ij).

## Supporting Information

Figure S1 **C-terminal domain of HopW1 forms complexes with actin in plants.** *N. benthamiana* was transiently transformed using Agrobacteria carrying HopW1 domains or full length tagged with HA. Complexes were immunoprecipitated with anti-HA agarose from dexamethasone-treated leaves and actin was detected by immunoblotting. -, not transformed control; N, HopW1-N-HA (dex:HopW1^Δ416–761^-HA); C, HopW1-C-HA (dex:HopW1^Δ19–417^-HA); W1, full length HopW1-HA (dex:HopW1^1–774^-HA). Asterisks (*) mark bands corresponding to monomeric HopW1-HA variants (HopW1 and HopW1-C are also detected in larger bands that may be dimers). Input was 3% of extract used for each IP. This experiment was repeated 3 times with similar results. Note that accumulation of HopW1-C is lower than other variants.
(TIF)

Figure S2 **HopW1 did not disrupt muscle F-actin.** Visualization of muscle F-actin. 0.5 µM of BSA (i), or 0.5 µM of HopW1-C (ii) was incubated with pre-assembled muscle F-actin (from chicken breast) for 1 h. Actin filaments were stained with TRITC-phalloidin and observed by epifluorescence microscopy. At least 100 actin filaments were measured from each sample and filament lengths were quantified (right panel). This experiment was repeated twice with similar results.
(TIF)

Figure S3 **Phenotypic effects of LatB on AALP:GFP and SPO:GFP localization.** (A) Example of microscopic analysis of the effect of LatB on AALP:GFP and SPO:GFP localization. Protoplasts from wild-type plants were transfected with AALP:GFP or SPO:GFP and incubated in 10 µM of LatB. Localization of AALP:GFP and SPO:GFP was examined using confocal fluorescence microscopy per time point, in two biological repeats. In the presence of LatB, the distribution patterns of the AALP:GFP and SPO:GFP showed similar punctate fluorescence patterns similar to those caused by HopW1. (B) Quantitation of the LatB-altered distribution patterns of AALP:GFP and SPO:GFP in Arabidopsis. Protoplasts were counted based on the distribution patterns in the presence and absence of LatB 12 h, 24 h, and 48 h after transfection from two biological repeats. Bars indicate SEM, *χ* ^2^ tests indicated that the distributions were significantly different between the wild type and LatB treatment at each time point for each marker protein fusion (*P*<0.0001, n≥30).
(TIF)

Table S1 **Bacterial strains.**
(DOCX)

Table S2 **Plasmids.**
(DOCX)

Text S1 **Contains supporting materials and methods and supporting references.**
(DOCX)

## References

[1] Jones JD, Dangl JL (2006) The plant immune system. Nature 444: 323–329. 17108957 10.1038/nature05286

[2] Sarkar SF, Gordon JS, Martin GB, Guttman DS (2006) Comparative genomics of host-specific virulence in Pseudomonas syringae. Genetics 174: 1041–1056. 16951068 10.1534/genetics.106.060996 PMC1602070

[3] Agrios GN (2005) Plant Pathology. San Diego: Academic Press.

[4] Abramovitch RB, Anderson JC, Martin GB (2006) Bacterial elicitation and evasion of plant innate immunity. Nat Rev Mol Cell Biol 7: 601–611. 16936700 10.1038/nrm1984 PMC2842591

[5] Block A, Alfano JR (2011) Plant targets for Pseudomonas syringae type III effectors: virulence targets or guarded decoys? Curr Opin Microbiol 14: 39–46. 21227738 10.1016/j.mib.2010.12.011 PMC3040236

[6] Jelenska J, Yao N, Vinatzer BA, Wright CM, Brodsky JL, et al (2007) A J domain virulence effector of Pseudomonas syringae remodels host chloroplasts and suppresses defenses. Curr Biol 17: 499–508. 17350264 10.1016/j.cub.2007.02.028 PMC1857343

[7] Xiang T, Zong N, Zou Y, Wu Y, Zhang J, et al (2008) Pseudomonas syringae effector AvrPto blocks innate immunity by targeting receptor kinases. Curr Biol 18: 74–80. 18158241 10.1016/j.cub.2007.12.020

[8] Lindeberg M, Cunnac S, Collmer A (2012) Pseudomonas syringae type III effector repertoires: last words in endless arguments. Trends Microbiol 20: 199–208. 22341410 10.1016/j.tim.2012.01.003

[9] Lee AH, Hurley B, Felsensteiner C, Yea C, Ckurshumova W, et al (2012) A bacterial acetyltransferase destroys plant microtubule networks and blocks secretion. PLoS Pathog 8: e1002523. 22319451 10.1371/journal.ppat.1002523 PMC3271077

[10] Shimada C, Lipka V, O'Connell R, Okuno T, Schulze-Lefert P, et al (2006) Nonhost resistance in Arabidopsis-Colletotrichum interactions acts at the cell periphery and requires actin filament function. Mol Plant-Microbe Interact 19: 270–279. 16570657 10.1094/MPMI-19-0270

[11] Miklis M, Consonni C, Bhat RA, Lipka V, Schulze-Lefert P, et al (2007) Barley MLO modulates actin-dependent and actin-independent antifungal defense pathways at the cell periphery. Plant Physiol 144: 1132–1143. 17449647 10.1104/pp.107.098897 PMC1914182

[12] Henty-Ridilla JL, Shimono M, Li J, Chang JH, Day B, et al (2013) The plant actin cytoskeleton responds to signals from microbe-associated molecular patterns. PLoS Pathog 9: e1003290. 23593000 10.1371/journal.ppat.1003290 PMC3616984

[13] Henty-Ridilla JL, Li J, Day B, Staiger CJ (2014) ACTIN DEPOLYMERIZING FACTOR4 regulates actin dynamics during innate immune signaling in Arabidopsis. Plant Cell 26: 340–352. 24464292 10.1105/tpc.113.122499 PMC3963580

[14] Kim H, Park M, Kim SJ, Hwang I (2005) Actin filaments play a critical role in vacuolar trafficking at the Golgi complex in plant cells. Plant Cell 17: 888–902. 15722471 10.1105/tpc.104.028829 PMC1069706

[15] Bhavsar AP, Guttman JA, Finlay BB (2007) Manipulation of host-cell pathways by bacterial pathogens. Nature 449: 827–834. 17943119 10.1038/nature06247

[16] Franco IS, Shuman HA (2012) A pathogen's journey in the host cell: Bridges between actin and traffic. BioArchitecture 2: 38–42. 22754628 10.4161/bioa.20422 PMC3383720

[17] Carabeo R (2011) Bacterial subversion of host actin dynamics at the plasma membrane. Cell Microbiol 13: 1460–1469. 21790944 10.1111/j.1462-5822.2011.01651.x PMC3174476

[18] Dunn JD, Valdivia RH (2010) Uncivil engineers: Chlamydia, Salmonella and Shigella alter cytoskeleton architecture to invade epithelial cells. Future Microbiol 5: 1210–1232. 10.2217/fmb.10.77 20722600

[19] Guo M, Tian F, Wamboldt Y, Alfano JR (2009) The majority of the type III effector inventory of Pseudomonas syringae pv. tomato DC3000 can suppress plant immunity. Mol Plant-Microbe Interact 22: 1069–1080. 19656042 10.1094/MPMI-22-9-1069 PMC2778199

[20] Guttman DS, Vinatzer BA, Sarkar SF, Ranall MV, Kettler G, et al (2002) A functional screen for the type III (Hrp) secretome of the plant pathogen Pseudomonas syringae. Science 295: 1722–1726. 11872842 10.1126/science.295.5560.1722

[21] Lee MW, Jelenska J, Greenberg JT (2008) Arabidopsis proteins important for modulating defense responses to Pseudomonas syringae that secrete HopW1-1. Plant J 54: 452–465. 18266921 10.1111/j.1365-313X.2008.03439.x

[22] Greenberg JT, Vinatzer BA (2003) Identifying type III effectors of plant pathogens and analyzing their interaction with plant cells. Curr Opin Microbiol 6: 20–28. 12615215 10.1016/s1369-5274(02)00004-8

[23] Thomas C, Tholl S, Moes D, Dieterle M, Papuga J, et al (2009) Actin bundling in plants. Cell Motil Cytoskeleton 66: 940–957. 19504571 10.1002/cm.20389

[24] Riedl J, Crevenna AH, Kessenbrock K, Yu JH, Neukirchen D, et al (2008) Lifeact: a versatile marker to visualize F-actin. Nat Methods 5: 605–607. 18536722 10.1038/nmeth.1220 PMC2814344

[25] Higaki T, Kutsuna N, Sano T, Kondo N, Hasezawa S (2010) Quantification and cluster analysis of actin cytoskeletal structures in plant cells: role of actin bundling in stomatal movement during diurnal cycles in Arabidopsis guard cells. Plant J 61: 156–165. 20092030 10.1111/j.1365-313x.2009.04032.x

[26] Smertenko AP, Deeks MJ, Hussey PJ (2010) Strategies of actin reorganisation in plant cells. J Cell Sci 123: 3019–3028. 20699356 10.1242/jcs.071126

[27] Senetar MA, Foster SJ, McCann RO (2004) Intrasteric inhibition mediates the interaction of the I/LWEQ module proteins Talin1, Talin2, Hip1, and Hip12 with actin. Biochem 43: 15418–15428. 15581353 10.1021/bi0487239

[28] Tian M, Chaudhry F, Ruzicka DR, Meagher RB, Staiger CJ, et al (2009) Arabidopsis actin-depolymerizing factor AtADF4 mediates defense signal transduction triggered by the Pseudomonas syringae effector AvrPphB. Plant Physiol 150: 815–824. 19346440 10.1104/pp.109.137604 PMC2689984

[29] Porter K, Shimono M, Tian M, Day B (2012) Arabidopsis Actin-Depolymerizing Factor-4 links pathogen perception, defense activation and transcription to cytoskeletal dynamics. PLoS Pathog 8: e1003006. 23144618 10.1371/journal.ppat.1003006 PMC3493479

[30] Samaj J, Baluska F, Voigt B, Schlicht M, Volkmann D, et al (2004) Endocytosis, actin cytoskeleton, and signaling. Plant Physiol 135: 1150–1161. 15266049 10.1104/pp.104.040683 PMC519036

[31] Vida TA, Emr SD (1995) A new vital stain for visualizing vacuolar membrane dynamics and endocytosis in yeast. J Cell Biol 128: 779–792. 7533169 10.1083/jcb.128.5.779 PMC2120394

[32] Zipfel C, Robatzek S, Navarro L, Oakeley EJ, Jones JDG, et al (2004) Bacterial disease resistance in Arabidopsis through flagellin perception. Nature 428: 764–767. 15085136 10.1038/nature02485

[33] Robatzek S, Chinchilla D, Boller T (2006) Ligand-induced endocytosis of the pattern recognition receptor FLS2 in Arabidopsis. Genes Dev 20: 537–542. 16510871 10.1101/gad.366506 PMC1410809

[34] Chinchilla D, Zipfel C, Robatzek S, Kemmerling B, Nurnberger T, et al (2007) A flagellin-induced complex of the receptor FLS2 and BAK1 initiates plant defence. Nature 448: 497–500. 17625569 10.1038/nature05999

[35] Sharfman M, Bar M, Ehrlich M, Schuster S, Melech-Bonfil S, et al (2011) Endosomal signaling of the tomato leucine-rich repeat receptor-like protein LeEix2. Plant J 68: 413–423. 21736652 10.1111/j.1365-313X.2011.04696.x

[36] Li J, Zhao-Hui C, Batoux M, Nekrasov V, Roux M, et al (2009) Specific ER quality control components required for biogenesis of the plant innate immune receptor EFR. Proc Natl Acad Sci U S A 106: 15973–15978. 19717464 10.1073/pnas.0905532106 PMC2747228

[37] Wang D, Weaver ND, Kesarwani M, Dong X (2005) Induction of protein secretory pathway is required for systemic acquired resistance. Science 308: 1036–1040. 15890886 10.1126/science.1108791

[38] Hatsugai N, Hara-Nishimura I (2010) Two vacuole-mediated defense strategies in plants. Plant Signal Behav 5: 1568–1570. 21512325 10.4161/psb.5.12.13319 PMC3115105

[39] Stevens JM, Galyov EE, Stevens MP (2006) Actin-dependent movement of bacterial pathogens. Nat Rev Microbiol 4: 91–101. 16415925 10.1038/nrmicro1320

[40] Shao F (2008) Biochemical functions of Yersinia type III effectors. Curr Opin Microbiol 11: 21–29. 18299249 10.1016/j.mib.2008.01.005

[41] Franklin-Tong VE, Gourlay CW (2008) A role for actin in regulating apoptosis/programmed cell death: evidence spanning yeast, plants and animals. Biochem J 413: 389–404. 18613816 10.1042/BJ20080320

[42] Büttner D, Bonas U (2003) Common infection strategies of plant and animal pathogenic bacteria. Curr Opin Plant Biol 6: 312–319. 12873524 10.1016/s1369-5266(03)00064-5

[43] Yarar D, Waterman-Storer CM, Schmid SL (2005) A dynamic actin cytoskeleton functions at multiple stages of clathrin-mediated endocytosis. Mol Biol Cell 16: 964–975. 15601897 10.1091/mbc.E04-09-0774 PMC545926

[44] Kandasamy MK, McKinney EC, Roy E, Meagher RB (2012) Plant vegetative and animal cytoplasmic actins share functional competence for spatial development with protists. The Plant cell 24: 2041–2057. 22589468 10.1105/tpc.111.095281 PMC3442586

[45] Deeks MJ, Cvrckova F, Machesky LM, Mikitova V, Ketelaar T, et al (2005) Arabidopsis group Ie formins localize to specific cell membrane domains, interact with actin-binding proteins and cause defects in cell expansion upon aberrant expression. New Phytol 168: 529–540. 16313636 10.1111/j.1469-8137.2005.01582.x

[46] Deeks MJ, Calcutt JR, Ingle EK, Hawkins TJ, Chapman S, et al (2012) A superfamily of actin-binding proteins at the actin-membrane nexus of higher plants. Curr Biol 22: 1595–1600. 22840520 10.1016/j.cub.2012.06.041

[47] Jelenska J, Hal JAv, Greenberg JT (2010) Pseudomonas syringae hijacks plant stress chaperone machinery for virulence. Proc Natl Acad Sci U S A 107: 13177–13182. 20615948 10.1073/pnas.0910943107 PMC2919979

[48] Jin JB, Kim YA, Kim SJ, Lee SH, Kim DH, et al (2001) A new dynamin-like protein, ADL6, is involved in trafficking from the trans-Golgi network to the central vacuole in Arabidopsis. Plant Cell 13: 1511–1526. 11449048 10.1105/TPC.000534 PMC139540

[49] Lee KH, Kim DH, Lee SW, Kim ZH, Hwang I (2002) In vivo import experiments in protoplasts reveal the importance of the overall context but not specific amino acid residues of the transit peptide during import into chloroplasts. Mol Cells 14: 388–397. 12521302

[50] Blanchoin L, Pollard TD, Mullins RD (2000) Interactions of ADF/cofilin, Arp2/3 complex, capping protein and profilin in remodeling of branched actin filament networks. Curr Biol 10: 1273–1282. 11069108 10.1016/s0960-9822(00)00749-1

[51] Kovar DR, Kuhn JR, Tichy AL, Pollard TD (2003) The fission yeast cytokinesis formin Cdc12p is a barbed end actin filament capping protein gated by profilin. J Cell Biol 161: 875–887. 12796476 10.1083/jcb.200211078 PMC2172974

[52] Lu H, Salimian S, Gamelin E, Wang G, Fedorowski J, et al (2009) Genetic analysis of acd6-1 reveals complex defense networks and leads to identification of novel defense genes in Arabidopsis. Plant J 58: 401–412. 19144005 10.1111/j.1365-313X.2009.03791.x PMC2727925

