# Supplementary figures and images for: HopW1 from Pseudomonas syringae Disrupts the Actin Cytoskeleton to Promote Virulence in Arabidopsis
Source: PLoS Pathog. 2014 Jun 26;10(6):e1004232. doi: 10.1371/journal.ppat.1004232 (PMC4072799; doi:10.1371/journal.ppat.1004232)

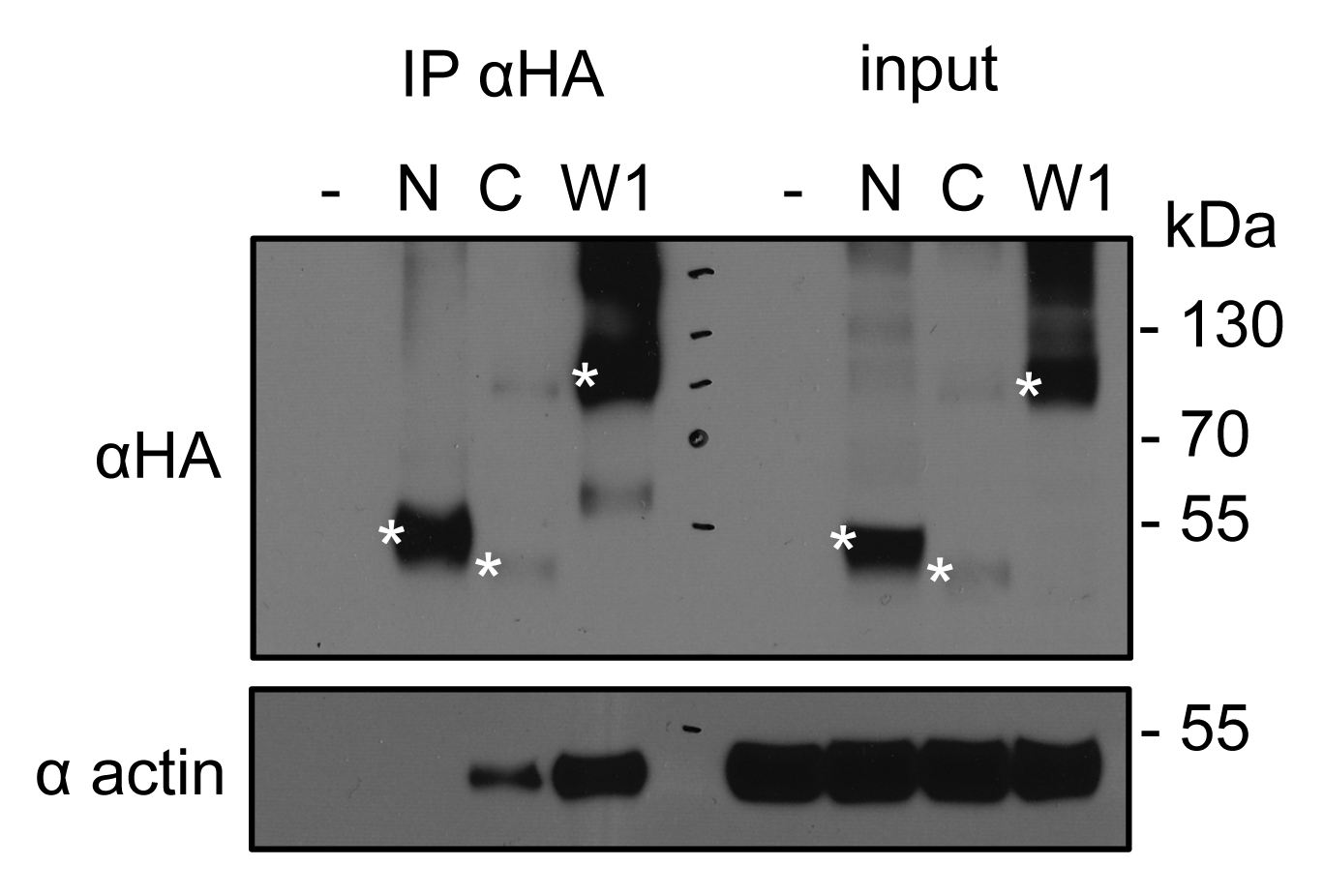

Supplement: Figure S1 — C-terminal domain of HopW1 forms complexes with actin in plants. N. benthamiana was transiently transformed using Agrobacteria carrying HopW1 domains or full length tagged with HA. Complexes were immunoprecipitated with anti-HA agarose from dexamethasone-treated leaves and actin was detected by immunoblotting. -, not transformed control; N, HopW1-N-HA (dex:HopW1Δ416–761-HA); C, HopW1-C-HA (dex:HopW1Δ19–417-HA); W1, full length HopW1-HA (dex:HopW11–774-HA). Asterisks (*) mark bands corresponding to monomeric HopW1-HA variants (HopW1 and HopW1-C are also detected in larger bands that may be dimers). Input was 3% of extract used for each IP. This experiment was repeated 3 times with similar results. Note that accumulation of HopW1-C is lower than other variants. (TIF) [file ppat.1004232.s001.tif]

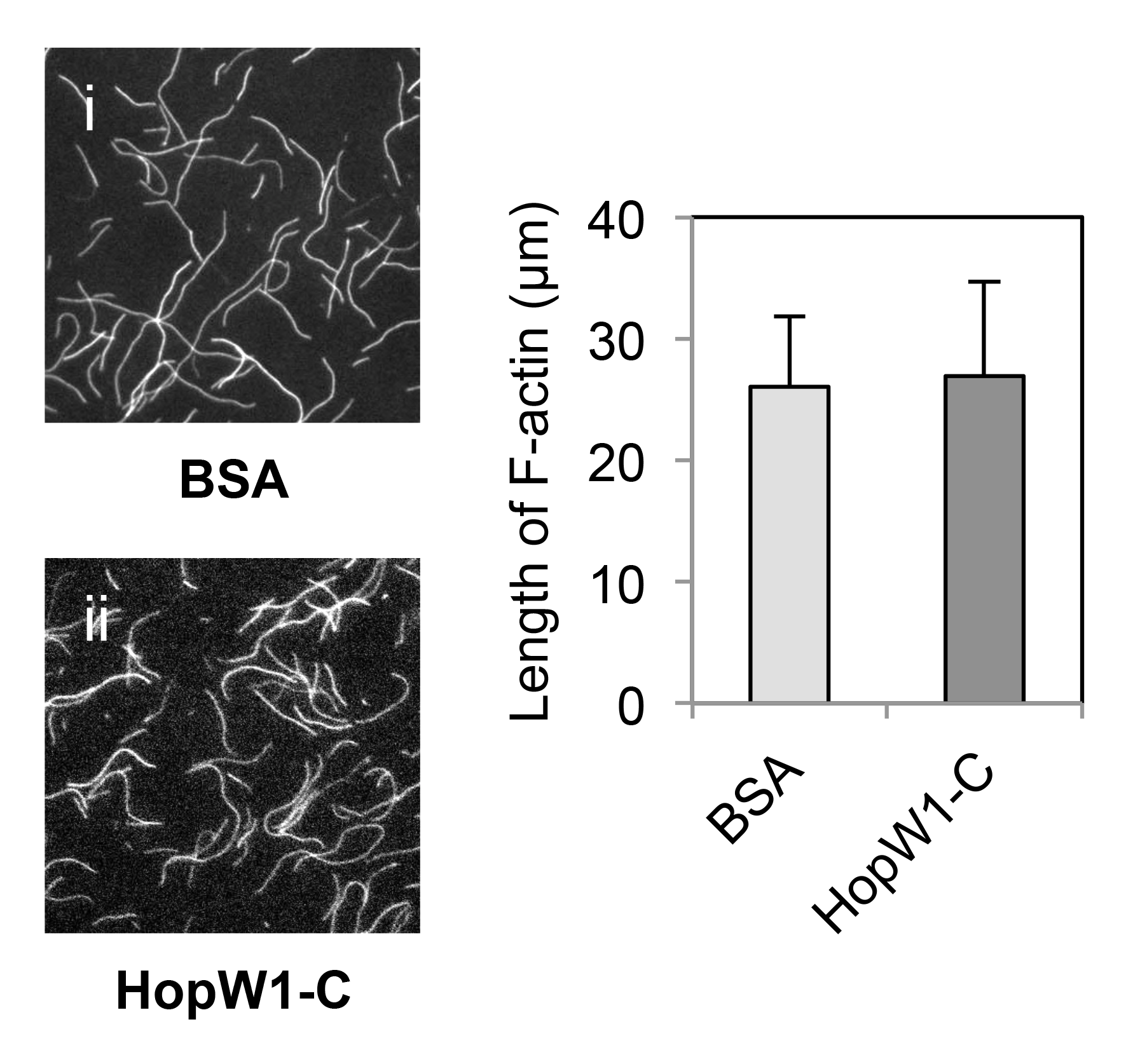

Supplement: Figure S2 — HopW1 did not disrupt muscle F-actin. Visualization of muscle F-actin. 0.5 µM of BSA (i), or 0.5 µM of HopW1-C (ii) was incubated with pre-assembled muscle F-actin (from chicken breast) for 1 h. Actin filaments were stained with TRITC-phalloidin and observed by epifluorescence microscopy. At least 100 actin filaments were measured from each sample and filament lengths were quantified (right panel). This experiment was repeated twice with similar results. (TIF) [file ppat.1004232.s002.tif]

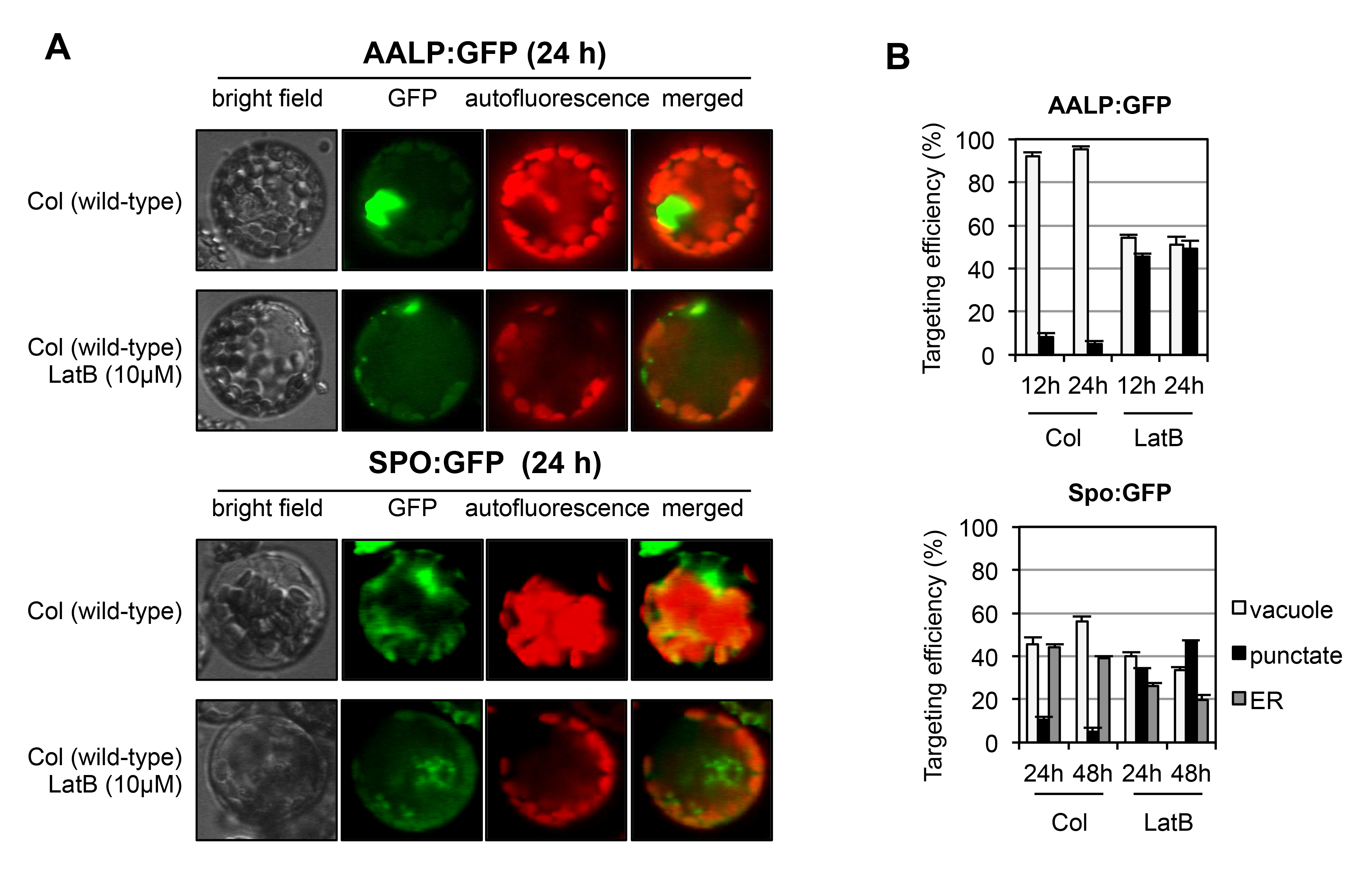

Supplement: Figure S3 — Phenotypic effects of LatB on AALP:GFP and SPO:GFP localization. (A) Example of microscopic analysis of the effect of LatB on AALP:GFP and SPO:GFP localization. Protoplasts from wild-type plants were transfected with AALP:GFP or SPO:GFP and incubated in 10 µM of LatB. Localization of AALP:GFP and SPO:GFP was examined using confocal fluorescence microscopy per time point, in two biological repeats. In the presence of LatB, the distribution patterns of the AALP:GFP and SPO:GFP showed similar punctate fluorescence patterns similar to those caused by HopW1. (B) Quantitation of the LatB-altered distribution patterns of AALP:GFP and SPO:GFP in Arabidopsis. Protoplasts were counted based on the distribution patterns in the presence and absence of LatB 12 h, 24 h, and 48 h after transfection from two biological repeats. Bars indicate SEM, χ 2 tests indicated that the distributions were significantly different between the wild type and LatB treatment at each time point for each marker protein fusion (P<0.0001, n≥30). (TIF) [file ppat.1004232.s003.tif]
