# Supplementary material for: HopW1 from Pseudomonas syringae Disrupts the Actin Cytoskeleton to Promote Virulence in Arabidopsis
Source: PLoS Pathog. 2014 Jun 26;10(6):e1004232. doi: 10.1371/journal.ppat.1004232 (PMC4072799; doi:10.1371/journal.ppat.1004232)
Supplement: Table S1 — Bacterial strains. (DOCX) [file ppat.1004232.s004.docx]

**Table S1. Bacterial Strains**

| Strain | Description | | Antibiotic^a)^ | | Reference |
| --- | --- | --- | --- | --- | --- |
| *Escherichia coli* | | | | | |
| DH5α | | F^-^ Φ80d*lac*ZΔ*M15* Δ(*lac*ZYA-*argF*)*U169* *endA1 recA1* *hsdR17* (r_K_^-^m_K_^+^) *deoR* *thi-*1 *supE44* λ^-^*gyrA96 relA1* | Nal^R^ | Invitrogen (Carlsbad, CA) | |
| DB3.1 | | F^-^ *gyrA462* *endA1 glnV44* Δ(sr1-*recA*) *mcrB* mrr *hsdS20*(r_B_^-^, m_B_^-^) *ara14* *galK2* *lacY1* *proA2* *rpsL20*(Sm^r^) *xyl5* Δ*leu* *mtl1* |  | Invitrogen (Carlsbad, CA) | |
| BL21 (DE3) | | F^-^ *ompT* *gal dcm lon hsdS_B_*(r_B_^-^ m_B_^-^) λ(DE3 [*lacI lacUV5*-T7 gene 1 *ind1 sam7 nin5*]) |  | Stratagene | |
| *Pseudomonas syringae* pv. *tomato* | | | | | |
| DC3000/  vector | | DC3000 carrying pME6012 | Rif^R^ /Tet^R^ | | [[9](#_ENREF_9)] |
| DC3000/  HopW1 | | DC3000 carrying pJJ72 (nptII:HopW1^1-774^-HA in pBAV179) |  | | [[9](#_ENREF_9)] |
| *Agrobacteriun tumefaciens* | | | | | |
| GV3101 | Used for *Agrobacterium*-mediated plant transformation | | Gm^R^/Rif^R^ | | [[10](#_ENREF_10)] |

a) Gm^R^, gentamycin resistance; Km^R^, kanamycin resistance; Nal^R^, nalidixic acid resistance; Rif^R^, rifampicin resistance; Sp^R^, spectinomycin resistance; Tet^R^, tetracycline resistance.
