# Supplementary material for: HopW1 from Pseudomonas syringae Disrupts the Actin Cytoskeleton to Promote Virulence in Arabidopsis
Source: PLoS Pathog. 2014 Jun 26;10(6):e1004232. doi: 10.1371/journal.ppat.1004232 (PMC4072799; doi:10.1371/journal.ppat.1004232)
Supplement: Table S2 — Plasmids. (DOCX) [file ppat.1004232.s005.docx]

**Table S2. Plasmids**

| Plasmid | Parent vector | Description | Antibiotic^a)^ | Reference |
| --- | --- | --- | --- | --- |
| CB302-3 |  | Binary plant expression vector (35S promoter) | Km^R^ /BASTA^R^ | [[11](#_ENREF_11)] |
| CAMBIA1300 |  | Binary plant expression vector | Km^R^/Hyg^R^ | http://www.cambia.org |
| GWB454 |  | Gateway binary plant expression vector (35S promoter, C-terminal mRFP) | Sp^R^/ Km^R^ | [[12](#_ENREF_12)] |
| DH51-GW-CFP |  | Gateway^TM^ CFP fusion vector for protoplast transformation | Amp^R^/Cm^R^ | Nottingham Arabidopsis Stock Centre |
| pET14b |  | T7 promoter and N-terminal His-tag | Amp^R^ | Invitrogen (Carlsbad, CA) |
| BAV154 | TA7001 | Gateway binary plant expression vector (*dex* promoter, C-terminal HA-tag) | Km^R^/ Cm^R^ /BASTA^R^ | [[13](#_ENREF_13)] |
| BAV103 | DONR207 | HopW1^1-774^ in DONR207 |  | [[9](#_ENREF_9)] |
| BAV179 | ME6012 | Gateway *P. syringae* vector (nptII promoter, C-terminal HA-tag) | Tet^R^/Cm^R^ | [[14](#_ENREF_14)] |
| JJ72 | BAV179 | nptII:HopW1^1-774^-HA in *P. syringae* vector |  | [[9](#_ENREF_9)], This study |
| pET:HopW1-C | pET14b | pET14b containing HopW1^407-774^ construct | Amp^R^ | This study |
| JJ31 | CB302-3 | CaMV 35S:HopW1-HA in a binary vector | Km^R^ /BASTA^R^ | This study |
| JJ74 | BAV154 | dex:HopW1-HA in a binary vector | Km^R^ /BASTA^R^ | This study |
| HopW1-N:HA | BAV154 | dex:HopW1^Δ416-761^-HA  (first 415 amino acids of HopW1 in a binary vector) | Km^R^ /BASTA^R^ | This study |
| HopW1-C:HA | BAV154 | dex:HopW1^Δ19-417^-HA  (amino acids 418-774 of HopW1 in a binary vector) | Km^R^ /BASTA^R^ | This study |
| 35S-mCHERRY | pCAMBIA1300 | CaMV 35S:mCHERRY in a binary vector | Km^R^/Hyg^R^ | This study |
| Lifeact-GFP | pMDC43 | CaMV 35S:Lifeact-GFP in a binary vector | Km^R^/Hyg^R^ | [[15](#_ENREF_15)] |
| HopW1:CFP | pDH51-GW-CFP | CaMV 35S:HopW1-CFP for protoplast transformation | Amp^R^ | This study |
| HopW1-RFP | pGWB454 | CaMV 35S:HopW1-mRFP in a binary vector | Sp^R^/ Km^R^ | This study |
| AALP:GFP | pUC | CaMV 35S:AALP-GFP for protoplast transformation | Amp^R^ | [[16](#_ENREF_16)] |
| SPO:GFP | pUC | CaMV 35S:SPO-GFP for protoplast transformation | Amp^R^ | [[16](#_ENREF_16)] |

a) Amp^R^, ampicillin resistance; BASTA^R^, BASTA (glufosinate ammonium) resistance; Cm^R^, chloroamphenicol resistance; Gm^R^, gentamycin resistance; Hyg^R^, hygromycin resistance; Km^R^, kanamycin resistance; Tet^R^, tetracycline resistance; Sp^R^, spectinomycin resistance.
