# Supplementary material for: HopW1 from Pseudomonas syringae Disrupts the Actin Cytoskeleton to Promote Virulence in Arabidopsis
Source: PLoS Pathog. 2014 Jun 26;10(6):e1004232. doi: 10.1371/journal.ppat.1004232 (PMC4072799; doi:10.1371/journal.ppat.1004232)
Supplement: Text S1 — Contains supporting materials and methods and supporting references. (DOCX) [file ppat.1004232.s006.docx]

**Text S1**

**Supporting Materials and Methods**

**Bacterial Strains, Plant Material and Growth Conditions.** All strains and plasmids used in this study are listed in Tables S1–S2. *Pseudomonas syringae* pv *tomato* DC3000 (*Pto*DC3000) and its derivatives (see Table S1), were grown on agar and liquid King’s broth [[1](#_ENREF_1)] with 34 μg/mL rifampicin at 30**°**C. *E. coli* DH5α and *E. coli* BL21 strains were grown on agar and liquid Luria-Bertani broth [[2](#_ENREF_2)] with appropriate antibiotics at 37**°**C. *Arabidopsis thaliana* accession Col and transgenic derivatives (Lifeact-GFP, kindly provided by Dr. Michael J. Deeks at the University of Durham [[3](#_ENREF_3)]; dex:HopW1-HA, stably transformed using JJ74 (Table S2)) were grown in soil for 2 to 3 weeks for assays of bacterial growth and protoplast isolation. Arabidopsis Col and Col/Lifeact-GFP grown on MS agar plates in a growth chamber for 6- to 8-d were used for actin quantification and endocytosis inhibition assays after bacterial infection. All Arabidopsis plants were grown under 16 h light, 8 h dark at 21**°**C. *N. benthamina* used for transient expression was grown for 3-4 weeks at 24**°**C with 16h light/8h dark cycle.

**Bacterial Growth Assay *in Planta.*** One ml blunt syringes were used to inoculate *Pto*DC3000 and its derivatives (see Table S1) at OD_600_ of 0.0001 in 10 mM MgSO_4_ and/or 10 μM LatB into leaves of 3-week old soil grown Arabidopsis. After infiltration, inoculated plants were covered and returned to the growth room. Bacterial populations were quantified three days post-inoculation from 3 samples each of which had 3 whole infected leaves. For statistical analysis, data from three independently performed experiments were analyzed together. Each inoculated leaf area was measured by Image J software, and then the leaves were homogenized in 1 mL of 10 mM MgSO_4_. Samples were serially diluted and enumerated by colony forming units after plating on KB agar with tetracycline (12.5 μg/mL).

**Immunoprecipitation and Western Blot Analysis.** Immunoprecipitation (IP) of HopW1-HA-containing complexes with anti-HA matrix (Roche) was done as described [[4](#_ENREF_4)]. HopW1-HA was immunoprecipitated from transgenic Arabidopsis (JJ74) 22 h after treatment with 0.3 μM dexamethasone (dex). *N. benthamiana* leaves were infiltrated with Agrobacterium GV3101 harboring HopW1-HA or its fragments. Tissue for IP was collected 36-38 h after Agroinfiltration with 35S construct (JJ31), or leaves were sprayed with 20 μM dex 22h after Agroinfiltration (JJ74, HopW1-C, HopW1-N) and collected 15h later. After IP, proteins were separated by SDS/PAGE and analyzed either by Coomassie blue staining and LC-MS/MS or by immunoblotting with anti-HA (1:1200, Covance) and anti-actin (1:1000, [[5](#_ENREF_5)]) monoclonal antibodies and secondary horseradish peroxidase-conjugated anti-mouse antibody (1:20,000, Thermo Fisher Scientific). Actin antibody was kindly provided by Dr. M V. Parthasarathy, Cornell University. Membranes were subsequently stained with Coomassie blue to control for loading using standard procedures for gels.

**Purification of Recombinant HopW1-C Protein.** The His-tagged HopW1-C (HopW1^407-774^) protein was produced in *Escherichia coli* BL21 (DE3) (Stratagene, USA). After isopropyl β-D-1-thiogalactopyranoside induction (1 μM, for 3 h) *E. coli* proteins were extracted in protein extraction buffer (50mM phosphate buffer, 300mM NaCl_2_, 2M urea, 10% glycerol, pH 8.0) and purified using Ni-NTA spin columns (Qiagen, USA). Protein extraction buffer was exchanged to protein buffer (50mM phosphate buffer, 300mM NaCl_2_, 5mM DTT, 10% glycerol, pH 8.0) using a Zeba™ desalting spin column (Thermo Scientific, USA) or dialysis.

**Arabidopsis Protoplast Isolation and Transformation.** Arabidopsis protoplasts were prepared from 2 - 3 week-old Arabidopsis leaves (Col, Col/Lifeact-GFP or Col/dex:HopW1). 0.5 − 1 mm leaf strips were digested in enzyme solution containing 1.5 % cellulase R10 (SERVA, Germany), 0.4 % macerozyme R10 (SERVA, Germany), 0.4 M mannitol, 20 mM KCl, 10 mM CaCl_2_, 0.1 % BSA, and 20 mM MES, pH 5.7. The digestions were continued for 3 h with gentle shaking in the dark. The resulting protoplasts were filtered through a 40 μm nylon filter and washed by centrifugation (100 × g, 5 min) and resuspended in buffer W5 (154 mM NaCl, 125 mM CaCl_2_, 5 mM KCl, and 2 mM MES, pH 5.7). The intact protoplasts were obtained using 20% sucrose-gradient centrifugation (500 × g, 5 min). Plasmids encoding HopW1*-*CFP or trafficking markers (SPO-GFP and AALP-GFP) (see Table S1) were introduced by polyethylene glycol mediated transformation [[6](#_ENREF_6),[7](#_ENREF_7)]. The expression of the fusion constructs was monitored by fluorescence microscopy at various time points after transformation.

**Confocal Microscopy.** For protein trafficking and endocytosis analysis, images were captured with an electron amplified CCD camera (ImagEM, Hamamatsu) and a confocal fluorescence microscope (Olympus DSU Spinning Disk Confocal System) using large pinhole, with the filter sets for GFP (ex:480/25 and em:525/40), CFP (ex:436/10 and em:470/30), and chlorophyll autofluorescence (ex:635/20 and em:685/40), respectively.

For actin cytoskeleton (marked by Lifeact-GFP) visualization in the presence of HopW1 in Arabidopsis protoplasts and *N. benthamiana* leaves or during infection of Arabidopsis cotyledons, a Zeiss LSM710 laser-scanning confocal microscope (Zeiss, Germany) was used. Fluorescence was visualized as follows: GFP excitation 488 nm/emission 505-530 nm ; mCherry/RFP: ex 561/em 570-620 nm; CFP: ex 405 nm/em 550-500 nm; and chlorophyll autofluorescence: ex 633/em 650-750 nm. Images (512x512 pixels scanning resolution in maximum speed mode) were taken using a LD C-Apochromat 40x/1.1 W Korr objective. Z-series optical sections of infected Arabidopsis and transformed *N. benthamiana* epidermal cells were captured. GFP and RFP or GFP and CFP fluorescence was acquired for the same field using a sequential acquisition mode.

Images were processed using Slidebook 5.0 (Intelligent Imaging Innovations, Inc., USA), ImageJ (http://rsb.info.nih.gov/ij) and Adobe Photoshop software.

**Muscle F-actin Disruption Assays.** For assays using chicken muscle actin, Ca-ATP actin was extracted from chicken muscle acetone powder (Pel-Freez Biologicals, Rogers, Arkansas) as described [[8](#_ENREF_8)]. Ca-ATP actin was converted to Mg-ATP actin by incubation with 0.1 volume of 0.5 mM MgCl_2_ and 2 mM EGTA for 2 min at room temperature prior to each experiment. F-actin was assembled from Mg-ATP-actin in buffer containing 10 mM Imidazole-HCl pH 7.0, 50 mM KCl, 1 mM EGTA, and 1 mM MgCl_2_ for 1 hour at room temperature. Preassembled muscle F-actin was incubated for 1h with HopW1-C or BSA and visualized as described for non-muscle actin.

**Supporting References**

1. King EO, Ward MK, Raney DE (1954) Two simple media for the demonstration of pyocyanin and fluorescin. J Lab Clin Med 44: 301-307.

2. Sambrook J, Fritsch EF, Maniatis T (1989) Molecular Cloning: A Laboratory Manual. NY: Cold Spring Harbor.

3. Deeks MJ, Cvrckova F, Machesky LM, Mikitova V, Ketelaar T, et al. (2005) Arabidopsis group Ie formins localize to specific cell membrane domains, interact with actin-binding proteins and cause defects in cell expansion upon aberrant expression. New Phytol 168: 529-540.

4. Jelenska J, Hal JAv, Greenberg JT (2010) Pseudomonas syringae hijacks plant stress chaperone machinery for virulence. Proc Natl Acad Sci U S A 107: 13177-13182.

5. Andersland JM, Fisher DD, Wymer CL, Cyr RJ, Parthasarathy MV (1994) Characterization of a monoclonal antibody prepared against plant actin. Cell Motil Cytoskeleton 29: 339-344.

6. Jin JB, Kim YA, Kim SJ, Lee SH, Kim DH, et al. (2001) A new dynamin-like protein, ADL6, is involved in trafficking from the trans-Golgi network to the central vacuole in Arabidopsis. Plant Cell 13: 1511-1526.

7. Lee KH, Kim DH, Lee SW, Kim ZH, Hwang I (2002) In vivo import experiments in protoplasts reveal the importance of the overall context but not specific amino acid residues of the transit peptide during import into chloroplasts. Mol Cells 14: 388-397.

8. Spudich JA, Watt S (1971) The regulation of rabbit skeletal muscle contraction. J Biol Chem 246: 4866-4871.

9. Lee MW, Jelenska J, Greenberg JT (2008) Arabidopsis proteins important for modulating defense responses to Pseudomonas syringae that secrete HopW1-1. Plant J 54: 452-465.

10. Koncz C, Schell J (1986) The promoter of TL-DNA gene 5 controls the tissue-specific expression of chimaeric genes carried by a novel type of Agrobacteriumbinary vector. Mol Gen Genet 204: 383-396.

11. Xiang C, Han P, Lutziger I, Wang K, Oliver DJ (1999) A mini binary vector series for plant transformation. Plant Mol Biol 40: 711-717.

12. Nakagawa T, Kurose T, Hino T, Tanaka K, Kawamukai M, et al. (2007) Development of series of gateway binary vectors, pGWBs, for realizing efficient construction of fusion genes for plant transformation. J Biosci Bioeng 104: 34-41.

13. Vinatzer BA, Teitzel GM, Lee MW, Jelenska J, Hotton S, et al. (2006) The type III effector repertoire of Pseudomonas syringae pv. syringae B728a and its role in survival and disease on host and non-host plants. Mol Microbiol 62: 26-44.

14. Vinatzer BA, Jelenska J, Greenberg JT (2005) Bioinformatics correctly identifies many type III secretion substrates in the plant pathogen Pseudomonas syringae and the biocontrol isolate P. fluorescens SBW25. Mol Plant-Microbe Interact 18: 877-888.

15. Smertenko AP, Deeks MJ, Hussey PJ (2010) Strategies of actin reorganisation in plant cells. J Cell Sci 123: 3019-3028.

16. Kim H, Park M, Kim SJ, Hwang I (2005) Actin filaments play a critical role in vacuolar trafficking at the Golgi complex in plant cells. Plant Cell 17: 888-902.
